# Supplementary figures and images for: CoExpPhylo – a novel pipeline for biosynthesis gene discovery
Source: BMC Genomics. 2025 Sep 22;26:807. doi: 10.1186/s12864-025-12061-3 (PMC12455792; doi:10.1186/s12864-025-12061-3)

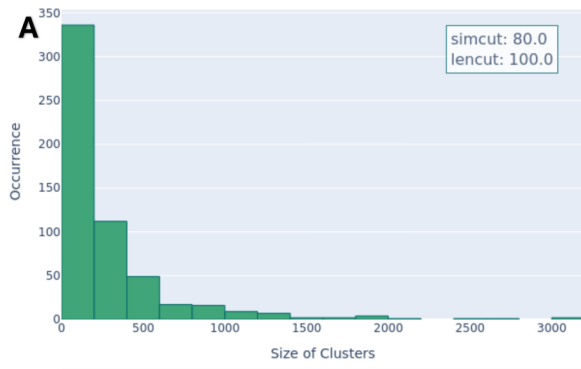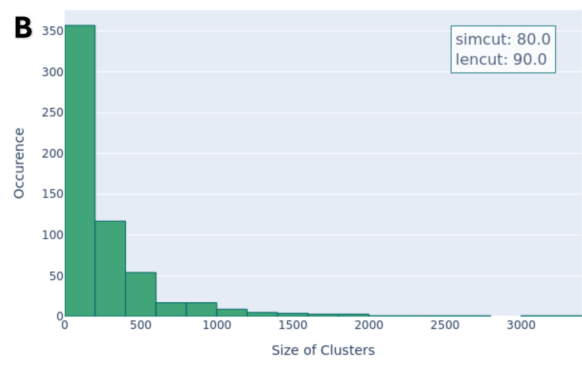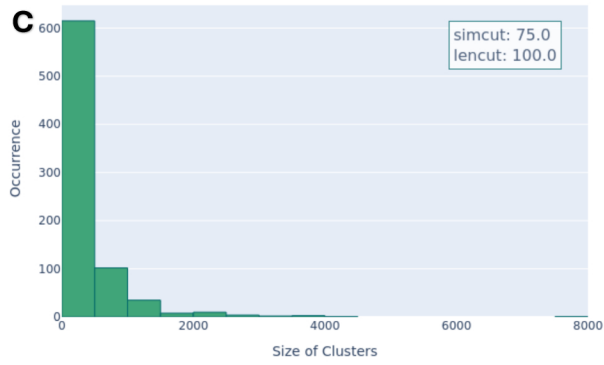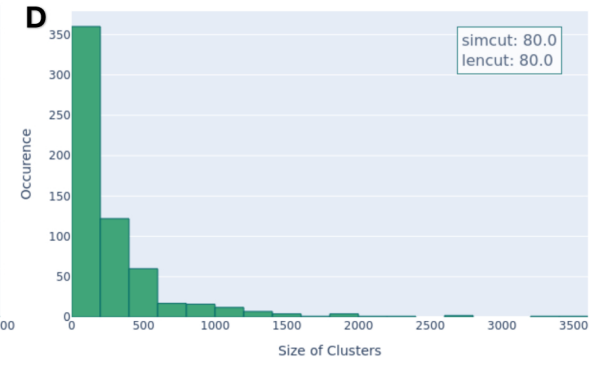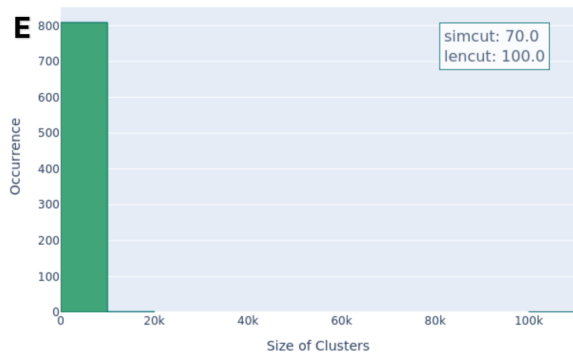

Supplement: Supplementary file 2 — Additional file 2 [file 12864_2025_12061_MOESM2_ESM.pdf]

## MAFFT

Tree scale: 0.1

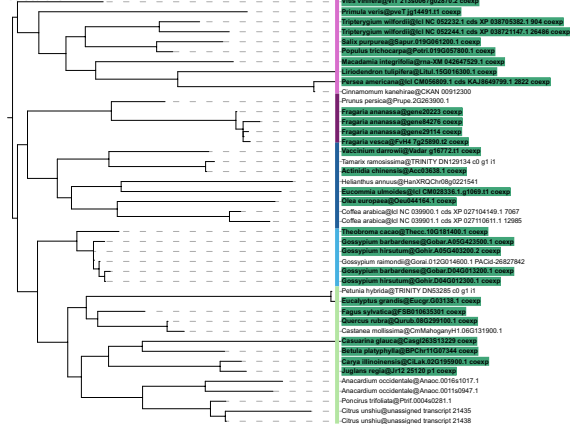

## MUSCLE

Tree scale: 0.1

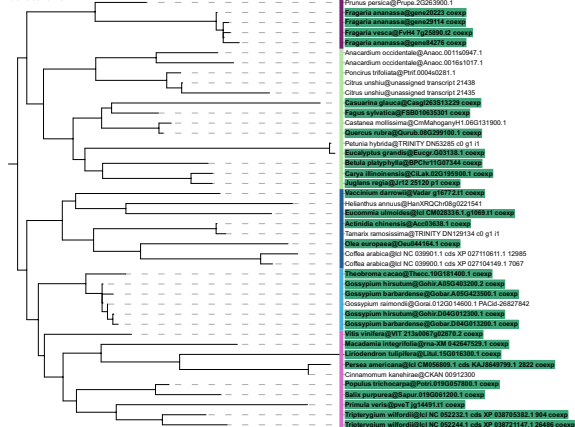

Supplement: Supplementary file 8 — Additional file 8 [file 12864_2025_12061_MOESM8_ESM.pdf]
