# Supplementary material for: CoExpPhylo – a novel pipeline for biosynthesis gene discovery
Source: BMC Genomics. 2025 Sep 22;26:807. doi: 10.1186/s12864-025-12061-3 (PMC12455792; doi:10.1186/s12864-025-12061-3)
Supplement: Supplementary file 9 — Additional file 9 [file 12864_2025_12061_MOESM9_ESM.pdf]

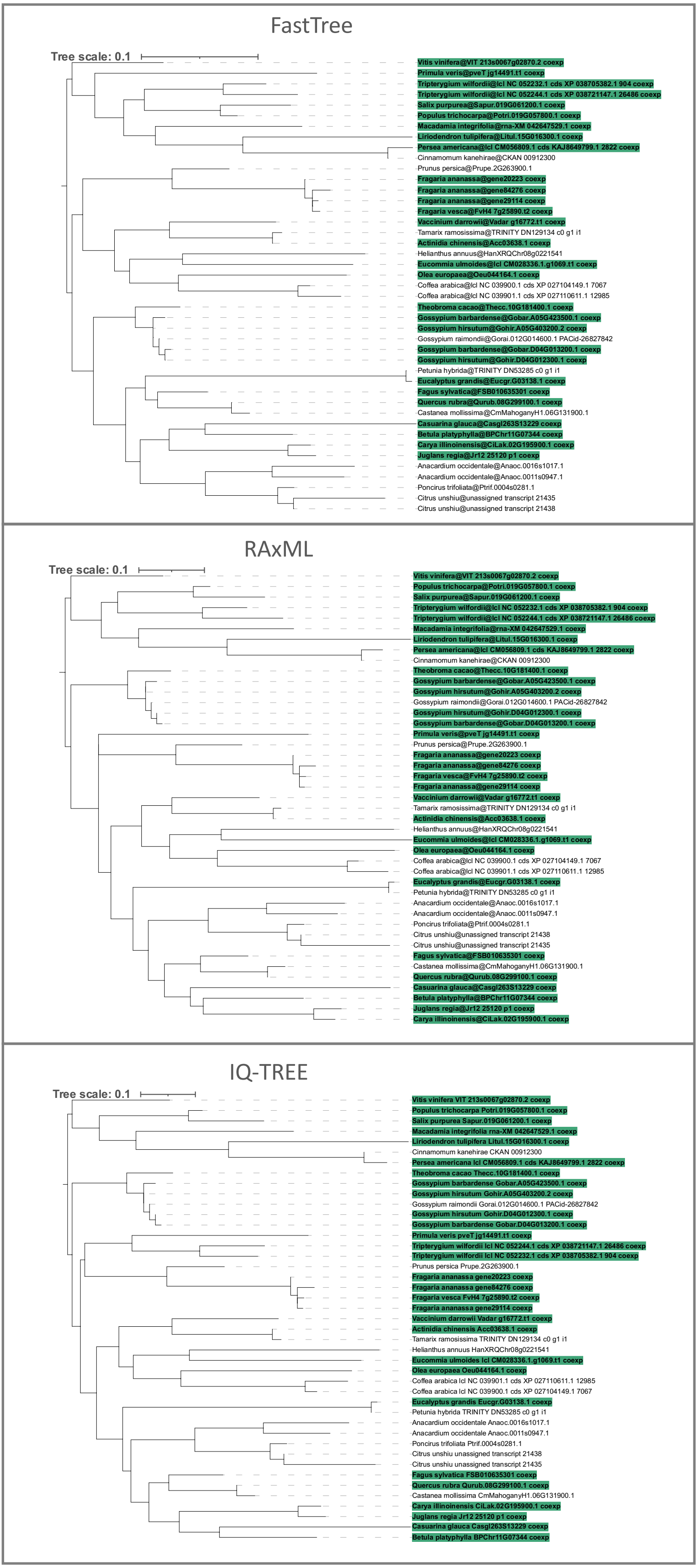
FastTree
Tree scale: 0.1
Vitis vinifera@VIT 213s0067g02870.2 coexp
Primula veris@pveT jg14491.t1 coexp
Tripterygium wilfordii@lcl NC 052232.1 cds XP 038705382.1 904 coexp
Tripterygium wilfordii@lcl NC 052244.1 cds XP 038721147.1 26486 coexp
Salix purpurea@Sapur.019G061200.1 coexp
Populus trichocarpa@Potri.019G057800.1 coexp
Macadamia integrifolia@rna-XM 042647529.1 coexp
Liriodendron tulipifera@Litul.15G016300.1 coexp
Persea americana@lcl CM056809.1 cds KAJ8649799.1 2822 coexp
Cinnamomum kanehirae@CKAN 00912300
Prunus persica@Prupe.2G263900.1
Fragaria ananassa@gene20223 coexp
Fragaria ananassa@gene84276 coexp
Fragaria ananassa@gene29114 coexp
Fragaria vesca@FvH4 7g25890.t2 coexp
Vaccinium darrowii@Vadar g16772.t1 coexp
Tamarix ramosissima@TRINITY DN129134 c0 g1 i1
Actinidia chinensis@Acc03638.1 coexp
Helianthus annuus@HanXRQChr08g0221541
Eucommia ulmoides@lcl CM028336.1.g1069.t1 coexp
Olea europaea@Oeu044164.1 coexp
Coffea arabica@lcl NC 039900.1 cds XP 027104149.1 7067
Coffea arabica@lcl NC 039901.1 cds XP 027110611.1 12985
Theobroma cacao@Thecc.10G181400.1 coexp
Gossypium barbardense@Gobar.A05G423500.1 coexp
Gossypium hirsutum@Gohir.A05G403200.2 coexp
Gossypium raimondii@Gorai.012G014600.1 PACid-26827842
Gossypium barbardense@Gobar.D04G013200.1 coexp
Gossypium hirsutum@Gohir.D04G012300.1 coexp
Petunia hybrida@TRINITY DN53285 c0 g1 i1
Eucalyptus grandis@Eucgr.G03138.1 coexp
Fagus sylvatica@FSB010635301 coexp
Quercus rubra@Qurub.08G299100.1 coexp
Castanea mollissima@CmMahoganyH1.06G131900.1
Casuarina glauca@Casgl263S13229 coexp
Betula platyphylla@BPChr11G07344 coexp
Carya illinoinensis@CiLak.02G195900.1 coexp
Juglans regia@Jr12 25120 p1 coexp
Anacardium occidentale@Anaoc.0016s1017.1
Anacardium occidentale@Anaoc.0011s0947.1
Poncirus trifoliata@Ptrif.0004s0281.1
Citrus unshiu@unassigned transcript 21435
Citrus unshiu@unassigned transcript 21438
RAxML
Tree scale: 0.1
Vitis vinifera@VIT 213s0067g02870.2 coexp
Populus trichocarpa@Potri.019G057800.1 coexp
Salix purpurea@Sapur.019G061200.1 coexp
Tripterygium wilfordii@lcl NC 052232.1 cds XP 038705382.1 904 coexp
Tripterygium wilfordii@lcl NC 052244.1 cds XP 038721147.1 26486 coexp
Macadamia integrifolia@rna-XM 042647529.1 coexp
Liriodendron tulipifera@Litul.15G016300.1 coexp
Persea americana@lcl CM056809.1 cds KAJ8649799.1 2822 coexp
Cinnamomum kanehirae@CKAN 00912300
Theobroma cacao@Thecc.10G181400.1 coexp
Gossypium barbardense@Gobar.A05G423500.1 coexp
Gossypium hirsutum@Gohir.A05G403200.2 coexp
Gossypium raimondii@Gorai.012G014600.1 PACid-26827842
Gossypium hirsutum@Gohir.D04G012300.1 coexp
Gossypium barbardense@Gobar.D04G013200.1 coexp
Primula veris@pveT jg14491.t1 coexp
Prunus persica@Prupe.2G263900.1
Fragaria ananassa@gene20223 coexp
Fragaria ananassa@gene84276 coexp
Fragaria vesca@FvH4 7g25890.t2 coexp
Fragaria ananassa@gene29114 coexp
Vaccinium darrowii@Vadar g16772.t1 coexp
Tamarix ramosissima@TRINITY DN129134 c0 g1 i1
Actinidia chinensis@Acc03638.1 coexp
Helianthus annuus@HanXRQChr08g0221541
Eucommia ulmoides@lcl CM028336.1.g1069.t1 coexp
Olea europaea@Oeu044164.1 coexp
Coffea arabica@lcl NC 039900.1 cds XP 027104149.1 7067
Coffea arabica@lcl NC 039901.1 cds XP 027110611.1 12985
Eucalyptus grandis@Eucgr.G03138.1 coexp
Petunia hybrida@TRINITY DN53285 c0 g1 i1
Anacardium occidentale@Anaoc.0016s1017.1
Anacardium occidentale@Anaoc.0011s0947.1
Poncirus trifoliata@Ptrif.0004s0281.1
Citrus unshiu@unassigned transcript 21438
Citrus unshiu@unassigned transcript 21435
Fagus sylvatica@FSB010635301 coexp
Castanea mollissima@CmMahoganyH1.06G131900.1
Quercus rubra@Qurub.08G299100.1 coexp
Casuarina glauca@Casgl263S13229 coexp
Betula platyphylla@BPChr11G07344 coexp
Juglans regia@Jr12 25120 p1 coexp
Carya illinoinensis@CiLak.02G195900.1 coexp
IQ-TREE
Tree scale: 0.1
Vitis vinifera VIT 213s0067g02870.2 coexp
Populus trichocarpa Potri.019G057800.1 coexp
Salix purpurea Sapur.019G061200.1 coexp
Macadamia integrifolia rna-XM 042647529.1 coexp
Liriodendron tulipifera Litul.15G016300.1 coexp
Cinnamomum kanehirae CKAN 00912300
Persea americana lcl CM056809.1 cds KAJ8649799.1 2822 coexp
Theobroma cacao Thecc.10G181400.1 coexp
Gossypium barbardense Gobar.A05G423500.1 coexp
Gossypium hirsutum Gohir.A05G403200.2 coexp
Gossypium raimondii Gorai.012G014600.1 PACid-26827842
Gossypium hirsutum Gohir.D04G012300.1 coexp
Gossypium barbardense Gobar.D04G013200.1 coexp
Primula veris pveT jg14491.t1 coexp
Tripterygium wilfordii lcl NC 052244.1 cds XP 038721147.1 26486 coexp
Tripterygium wilfordii lcl NC 052232.1 cds XP 038705382.1 904 coexp
Prunus persica Prupe.2G263900.1
Fragaria ananassa gene20223 coexp
Fragaria ananassa gene84276 coexp
Fragaria vesca FvH4 7g25890.t2 coexp
Fragaria ananassa gene29114 coexp
Vaccinium darrowii Vadar g16772.t1 coexp
Actinidia chinensis Acc03638.1 coexp
Tamarix ramosissima TRINITY DN129134 c0 g1 i1
Helianthus annuus HanXRQChr08g0221541
Eucommia ulmoides lcl CM028336.1.g1069.t1 coexp
Olea europaea Oeu044164.1 coexp
Coffea arabica lcl NC 039901.1 cds XP 027110611.1 12985
Coffea arabica lcl NC 039900.1 cds XP 027104149.1 7067
Eucalyptus grandis Eucgr.G03138.1 coexp
Petunia hybrida TRINITY DN53285 c0 g1 i1
Anacardium occidentale Anaoc.0016s1017.1
Anacardium occidentale Anaoc.0011s0947.1
Poncirus trifoliata Ptrif.0004s0281.1
Citrus unshiu unassigned transcript 21438
Citrus unshiu unassigned transcript 21435
Fagus sylvatica FSB010635301 coexp
Quercus rubra Qurub.08G299100.1 coexp
Castanea mollissima CmMahoganyH1.06G131900.1
Carya illinoinensis CiLak.02G195900.1 coexp
Juglans regia Jr12 25120 p1 coexp
Casuarina glauca Casgl263S13229 coexp
Betula platyphylla BPChr11G07344 coexp
